# Perceived ambidextrous leadership and nurses’ mental health: a work-family perspective

**DOI:** 10.1186/s12912-024-02090-w

**Published:** 2024-07-23

**Authors:** Jiaqi Yan, Yuefeng Lian, Liangxing He

**Affiliations:** 1 School of Business Administration, Northeastern University, Shenyang, China; 2 Business School, Nankai University, Tianjin, China; 3 School of Business Administration, Nanjing University of Finance & Economics, Nanjing, China; 4 Innovation & Entrepreneurship Research Center, Nanjing University of Finance & Economics, Nanjing, China

**Keywords:** Nurses; Perceived ambidextrous leadership, Work-to-family enrichment, Mental health, Work climate for sharing family concerns

## Abstract

**Background:**

Nurses are particularly at risk from stress-related problems and face high mental health problems during the COVID-19 pandemic. It is critical to pay attention to their mental health status and determine which factors are positively associated with nursing staff mental health from the perspective of work-family. The purpose of this paper is to investigate the impact of perceived ambidextrous leadership on nurses’ mental health mediated by work-to-family enrichment and moderated by work climate for sharing family concerns.

**Methods:**

One time-lagged study with three waves was conducted. A total of 358 questionnaires were distributed to registered nurses working at 10 hospitals in Guangzhou, China, and 265 valid questionnaires were returned. The quantitative approach to test hypotheses involves hierarchical regression analyses, the bootstrapping method and the simple slope test.

**Results:**

The research indicated that (a) perceived ambidextrous leadership had a positive influence on nurses’ work-to-family enrichment; (b) nurses’ work-to-family enrichment mediated the relationship between perceived ambidextrous leadership and nurses’ mental health; (c) work climate for sharing family concerns moderated the relationship between perceived ambidextrous leadership and nurses’ work-to-family enrichment.

**Conclusion:**

Nursing supervisors’ ambidextrous leadership interacted with work climate for sharing family concerns benefit the conservation of nurses’ personal resources, which in turn facilitates nurses’ work-to-family enrichment and improve their mental health.

## Introduction

During the COVID-19 pandemic, worldwide health systems are overwhelmed, accompanied by shortages of medical resources. Nurses undertake high-intensity work and are liable to extend their working hours, which results in the experience of role overload [1]. In combination with traditional features of the nursing profession (e.g., shiftwork and flexibility of work scheduling), it is unattainable for nurses to consider their work and family as separate domains [2, 3]. Nurses are confronted with more strain and passive outcomes from work-family interference compared to other occupations [4, 5]. Work-family issues during the COVID-19 pandemic seriously trouble nurses, which further leads to stress burnout and psychological unhealth [3, 6, 7].

Considering the potential poor mental health condition of the nursing profession during the COVID-19 pandemic [3, 8–12], work-to-family enrichment may help relieve the pressure. However, there is limited knowledge concerning work-to-family enrichment among nurses in previous research [13–15]. Work-family enrichment, including work-to-family enrichment and family-to-work enrichment, refers to the extent to which experiences in the family/work role can improve the quality of life in the other role [16]. A meta-analysis including 67 studies lends support to the notion that work-to-family enrichment contributes to health conditions [17]. Thus, we focus on work-to-family enrichment, considering pressure mainly caused by heavy work and prob in relation to nurses’ mental health.

Bakker et.al. [18] noted that a resource gain spiral can be attributed to work-to-family enrichment. Based on job demands-resources theory, leadership is considered to have a direct impact on employees’ job resources and personal resources [19]. That is, the influence of leadership can be a significant antecedent of work-to-family enrichment. Nevertheless, the previous study merely focuses on the effect of single leadership on work-to-family enrichment in static situations, neglecting the subsistence of the combination of different leadership types in a dynamic management environment [13, 20–22]. In the last decade, theorists have proposed one new leadership type termed ambidextrous leadership, combining two complementary leadership behaviors, that is, closing leader behaviors, which include establishing routines and controlling target achievement, and opening leader behaviors, which comprise motivating subordinates to take risks and encouraging different ways to achieve job tasks [23]. From a dialectical perspective, health organizations are complex in nature and are confronted with changing and contradictory requirements that resemble those of other industries [23]. These requirements, especially existing in the work-family interface, are constantly contradictory and contain fundamentally different logics and patterns, which need to be coordinated and integrated by supervisors with limited organizational resources [24]. Ambidextrous leadership, as a combination of complementary leadership, enables leaders to discharge contradictory tasks and manage complicated situations [25]. With regard to the work-family interface, ambidextrous leadership assists subordinates with their work-family role overlap issues and provides them with heterogeneous resources for the promotion of work-to-family enrichment. Therefore, this research aims to examine whether ambidextrous leadership, as perceived by followers, enhances work-to-family enrichment in nurses.

The work-home resources model posits that different dimensions of resources (contextual and personal resources, volatile and structural resources, key resources and macro resources) influence work-family enrichment through different mechanisms [26]. Organizational work climate, as a specific aspect of employees’ social context, refers to the shared perceptions of collective employees concerning their perceptions of the organizational environment [27]. Based on the conservation of resources theory [28, 29], organizational work climate can be regarded as a source of resources, which in turn facilitates employees’ work-to-family enrichment by providing work-related resources [26]. Specifically, a few studies have identified the influence of work climate for sharing family concerns on the work-family interface [30–33]. Thus, this paper also investigates the moderating role of work climate for sharing family concerns on the relationship between perceived ambidextrous leadership and work-family enrichment.

## Theoretical background and hypotheses

### Perceived **ambidextrous leadership and work-to-family enrichment**

Based on the work-home resources model [26], work-related resources may have a positive impact on the family domain. Supervisors can affect subordinates’ working environment by providing a large proportion of work-related resources and demands for them [34]. To date, a few studies have identified the influence of leader-member exchange, authentic leadership, servant leadership and empowering leadership on work-to-familyenrichment [13, 20–22]. Concerning nursing management,Cortese et.al. [35] also regard workplace leadership as helping nurses manage the relationship with their families. In addition, during the COVID-19 pandemic, nurses are under massive physical and emotional strain [36]. It is, therefore, more difficult to enhance nurses’ work-to-family enrichment, given contradictory requirements between the family domain and work domain [37].

Ambidextrous leadership is usually viewed as one leadership style that can address changing and contradictory requirements. Drawing from the work-home resources model [26], work-related resources derived from ambidextrous leadership can contribute to a gain spiral of personal resources and further improve the accumulation of family-related resources, which facilitates work-to-family enrichment. Specifically, ambidextrous leadership consists of opening leadership and closing leadership. Openning leadership provides room for employees to think and work independently [23, 25]. With job autonomy, which is one core dimension of job design [38], employees are endowed with a set of psychological capital, including psychological empowerment [39] and psychological ownership [40]. These various work domain resources further yield personal resources such as self-efficacy [41, 42], self-esteem [43] and vitality [42], which in turn improve the quality of life in the family domain. Closing leadership can reduce the variance of nurse behaviors and provide specific guidelines for uniform task attainment [23, 25]. When subordinates receive clear information about the job goal and superior’s constant feedback during the work, role clarity comes into being [44, 45]. Accordingly, individuals’ cognitive and physical demands about job design are reduced, which leads to the accumulation of personal resources [28, 29]. As a result, these extra personal resources can be devoted to the other domain, the family domain, in which the quality of life is improved [26]. Therefore, the current research proposes the following hypothesis:

#### Hypothesis 1

Perceived ambidextrous leadership is positively related to nurses’ work-to-family enrichment.

### The mediating role of work-to-family enrichment between perceived ambidextrous leadership and mental health among nurses

Nurses’ mental health care is an increasingly critical topic and a huge challenge in nursing management, particularly during the COVID-19 pandemic [8, 9, 46]. For instance, a survey of 1257 nurses in China found that over half of the participants experienced negative psychological emotions when treating patients exposed to COVID-19 [47]. In addition, many studies have indicated that nurses are one of the groups experiencing job burnout due to role overload and high work stress [48, 49], so they usually suffer from depression and anxiety [49].

Brummelhuis and Bakker [26] posit that work-to-family enrichment leads to the generation of personal resources (including physical, psychological, intellectual, affective, and capital resources) that are proximate to the self, contrary to contextual resources related to the special domain. These personal resources can be invested in nonwork-related activities such as leisure and, in turn, may benefit individual psychological health and general well-being. As studied by a meta-analysis [50], work-to-family enrichment is positively associated with physical and mental health. The findings are consistent with other similar studies [17]. Integrating Hypothesis 1, we regard perceived ambidextrous leadership as beneficial for nurses’ work-to-family enrichment and the maintenance of mental health. Therefore, we propose the following hypothesis:

#### Hypothesis 2

The work-to-family enrichment of nurses mediates the relationship between perceived ambidextrous leadership and mental health, that is, specifically, depression and anxiety.

### The moderating role of work climate for sharing family concerns

The work-home resources model [26] accentuates that social support, as the contextual resource provided by significant individuals within certain work environments, can influence the work-family enrichment process. Social information processing theory posits “the main argument is that there is some substitutability among cues such that effects can be communicated using some cues at the neglect of others” [51] (p. 3). Walther regards proximal social cues can substitute for distal social cues [51]. The work climate for sharing family concerns, as one specific aspect of workplace social support, refers to the organizational climate that encourages employees to share their concerns about the family domain within their work domain role [30, 52]. This construct has been found to directly and indirectly influence individual work and family life [30–33]. Moreover, in line with contingency theory [53], with internal fit among key components within an organization, such as individual leadership behavior and organizational environment, a coherent ensemble comes into being, within which originally independent elements interact interdependently. More specifically, leadership behavior has a distinct influence on subordinates depending on the situational variables in the organization [54].

**Fig. 1:**
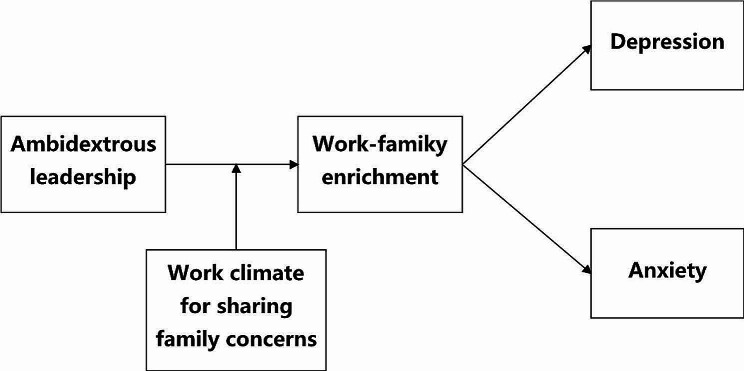
Theoretical hypothesis model

Therefore, we assume that the work climate for sharing family concerns moderates the relationship between perceived ambidextrous leadership and nurses’ work-to-family enrichment. When the work climate is characterized by sharing family concerns, the individual tends to discuss family concerns with peers. Compared with interactions with leaders, interpersonal relationships among colleagues are characterized by a greater sense of informality and ease, devoid of hierarchical power dynamics [55]. Colleagues engage in discussions encompassing a wide range of subjects, including familial matters, professional endeavors, and personal experiences [56]. Consequently, employees are more inclined to express their thoughts and emotions to their colleagues, thereby receiving a heightened level of emotional support from them as compared to their leaders. According to social information processing theory, colleagues can be viewed as proximal social cues and leadership can be viewed as distal social cues [51]. Accordingly, a substitution effect of colleagues appears with regard to the impact of leadership on nurses’ work-to-family enrichment. Specifically, colleagues serve as a source of resources for employees. While sharing feelings and concerns with colleagues about the family role and receiving feedback, individuals gain colleagues’ social support. These contextual resources can help them manage work-family issues [57] and may further enhance work-to-family enrichment [33]. This substitution effect will, in turn, lessen the influence of the superior’s leadership behavior on subordinates. Therefore, we propose the following hypothesis. Figure 1 shows our overall conceptual model.

#### Hypothesis 3

A work climate for sharing family concerns moderates the relationship between perceived ambidextrous leadership and nurses’ work-to-family enrichment. When the work climate for sharing family concerns is higher, the effect of perceived ambidextrous leadership on nurses’ work-to-family enrichment is weaker.

## Method

### Participants and data collection

The participants for the current study were registered nurses working at 10 hospitals in Guangzhou, China, for the past 12 months. This study excludes nurses who are no longer engaged in clinical nursing work. Data are gathered through convenience sampling. Among the hospitals, 2 hospitals had a capacity of less than 100 beds, 2 hospitals had a capacity of 100–250 beds, and 6 hospitals had a capacity of over 250 beds.

Data were collected from August 2021 to December 2021. First, we obtained approval to conduct the survey from the 10 directors of the target hospitals. Second, each nurse was provided with a questionnaire and a return sealed envelope and told only for academic purposes. To control the concern of common method bias, a time-lagged design was adopted with a 2-month interval. A total of 358 participants rated the demographic information, ambidextrous leadership, and work climate for sharing family concerns. Two months later, 295 of 358 participants rated work-to-family enrichment. Another two months later, 271 of 295 participants rated their mental health. We matched the three-wave survey by a consistent and independent code and received 265 final usable responses.

The sociodemographic characteristics of the nurses are indicated in Table 1. Of the 265 respondents, 27.92% were male, and 72.08% were female. A total of 35.85% were aged 18 years old to 25 years old, and 31.32% were aged 26 years old to 30 years old. A total of 60.38% possessed a bachelor’s degree, and 63.40% were married. A total of 33.21% had one child, and 16.60% had two children. A total of 44.91% reported monthly salaries ranging from 5001 yuan to 10,000 yuan. We found no significant differences in the demographic variables between the full respondents and partial respondents.

**Table 1:** Sociodemographic characteristics of nurses

Variables	*N*	Percentage
Gender		
Male	74	27.92%
Female	191	72.08%
Age		
18–25	95	35.85%
26–30	83	31.32%
31–40	52	19.62%
41–50	29	10.95%
51–60	6	2.26%
Education background		
High school	10	3.77%
Associate degree	72	27.17%
Bachelor’s degree	160	60.38%
Master’s/doctoral degree	23	8.68%
Marital status		
Not married	97	36.60%
Married	168	63.40%
Numbers of children		
0	131	49.43%
1	88	33.21%
2	44	16.60%
3	2	0.75%
Family month income		
0-5000	44	16.60%
5001–10,000	119	44.91%
10,001–20,000	70	26.42%
20,001–30,000	19	7.17%
30,001–40,000	5	1.89%
40,001–50,000	8	3.02%

### Measure

#### Perceived ambidextrous leadership

We adopted Zacher and Rosing’s [25] 14-item scale. Seven items belong to the opening leadership dimension, and the other 7 items belong to the closing leadership dimension. We adopted Zacher and Rosing’s scale because it had been tested with good reliability and validity in the Chinese context (e.g., Ma et.al. [58]). Sample items are “My supervisor pays attention to uniform task accomplishment” and “My supervisor gives possibilities for independent thinking and acting”. The Cronbach’s alpha was 0.906.

#### Work-to-family enrichment

We adopted Carlson et.al.’s [59] 9-item scale. Sample items are “the involvement in my work helps me to gain knowledge and this helps me be a better family member” and “the involvement in my work helps me acquire skills and this helps me be a better family member”. The Cronbach’s alpha was 0.950.

#### Work climate for sharing family concerns

We adopted Kossek et.al.’s [52] 3-item scale. Sample items are “In my department, it is generally accepted that people might share concerns about their family” and “In my department, it is generally accepted that people can get advice on how to deal with family issues”. The Cronbach’s alpha was 0.842.

#### Mental health

We adopted the Löwe et.al.’s [60] clinically validated PHQ-4 scale to measure mental health disease. Sample items are “In the last week, how often have you been bothered by little interest or pleasure in doing things” and “In the last week, how often have you been bothered by feeling down, depressed, or hopeless”. The Cronbach’s alpha was 0.917.

#### Control variables

We followed prior studies on mental health during the COVID-19 pandemic [61, 62] to control for gender (1 = male, 2 = female), age (1 = 18–25, 2 = 26–30, 3 = 31–40, 4 = 41–50; 5 = 51–60), educational background (1 = high school, 2 = associate degree, 3 = bachelor’s degree, 4 = master’s/doctoral degree), marital status (1 = not married, 2 = married), number of children (1 = 0 child, 2 = 1 child, 3 = 2 children, 4 = 3 children) and family monthly income (1 = 0-5000 yuan, 2 = 10,001–20,000 yuan, 3 = 20,001–30,000 yuan, 4 = 30,001–40,000 yuan, 5 = 40,001–50,000 yuan).

## Results

### Descriptive statistics and correlations among variables

We used SPSS 21.0 to analyzed the correlations among variables. Table 2 indicates the mean, SD and correlations among all the variables. The mean scores for perceived ambidextrous leadership, work-to-family enrichment, work climate for sharing family concerns and mental health disease were 3.871 (SD = 0.662), 3.894 (SD = 0.802), 3.442 (SD = 0.744) and 1.947 (SD = 0.857), respectively. Perceived ambidextrous leadership was positively correlated with work-to-family enrichment. Mental health was negatively correlated with perceived ambidextrous leadership and work-to-family enrichment.

**Table 2:** **Means, SD and correlation** Note: *n* = 265; ^***^p < 0.001, ^**^p < 0.01, ^*^p < 0.05

	Mean	SD	1	2	3	4	5	6	7	8	9	10
1.*gender*	1.721	0.45										
2.*age*	2.000	1.204	0.113^*^									
3.*education background*	2.740	0.666	-0.105^*^	-0.223^***^								
4.*marital status*	1.634	0.483	0.068	0.387^***^	-0.097							
5.*children*	0.687	0.771	0.064	0.326^***^	-0.174^***^	0.607^***^						
6.*family month income*	2.478	1.086	-0.066	-0.134^**^	0.332^***^	0.087	0.100					
7.*hospital size*	2.469	0.751	-0.099	-0.216^***^	0.225^***^	-0.126^**^	-0.231^***^	0.237^***^				
8.*Ambidextrous leadership*	3.871	0.662	0.057	-0.029	-0.033	0.121^**^	0.096	0.057	0.089			
*9.Work-to-family enrichment*	3.894	0.802	0.099	0.025	0.005	0.170^***^	0.036	0.056	0.079	0.529^***^		
10.*Work-climate for sharing concerns*	3.442	0.744	0.053	0.116^*^	-0.068	0.125^**^	0.119^*^	-0.067	0.048	0.445^***^	0.517^***^	
11. *Mental health disease*	1.947	0.857	0.035	-0.009	-0.057	-0.008	0.084	0.059	-0.032	-0.142^**^	-0.213^***^	0.096

Note: *n* = 265; ^***^p < 0.001, ^**^p < 0.01, ^*^p < 0.05.

### Common method analysis

Considering that all variables are evaluated by employees, common method bias may be a concern. A time-lagged design was adopted to reduce common method bias. In addition, we added one unmeasured latent method factor by using MPLUS 7.4 when running confirmatory factor analysis and analysis the changes of model fit indicators (ΔCFI = 0, ΔTLI = 0.01, ΔRMSEA = 0.01, ΔSRMR = 0). Therefore, common method variance is not a serious concern in our study.

### Confirmatory factor analysis

We adopted MPLUS 7.4 to conduct a CFA test to evaluate the distinctness of variables. Table 3 indicates a four-factor model (x2/df = 2.950; CFI = 0.923; TLI = 0.905; RMSEA = 0.090; SRMR = 0.049) meets the indices of global model fit and fits better than the other models.

**Table 3:** Measure model

Model	Variables	X2/df	CFI	TLI	RMSEA	SRMR
One-factor	Perceived ambidextrous leadership + Work-climate for sharing concerns + Work-to-family enrichment + Mental health	14.070	0.501	0.424	0.222	0.164
Two-factor	Perceived ambidextrous leadership + Work-climate for sharing concerns; Work-to-family enrichment + Mental health	11.657	0.597	0.531	0.201	0.207
Three-factor	Perceived ambidextrous leadership + Work-climate for sharing concerns; Work-to-family enrichment; Mental health	4.687	0.863	0.838	0.118	0.078
Four-factor	Perceived ambidextrous leadership; Work-to-family enrichment; Work-climate for sharing concerns; Mental health	2.950	0.923	0.905	0.090	0.049

### Hypothesis test

We used SPSS 21.0 to test all the hypotheses. Hypothesis 1 predicted a positive relationship between perceived ambidextrous leadership and work-to-family enrichment. Table 4 shows that perceived ambidextrous leadership was positively related to work-to-family enrichment (Model 2, β = 0.596, *p* = 0.000). Thus, Hypothesis 1 was supported.

**Table 4:** Results of regression analyses

	Work-to-family enrichment			Mental health disease		
	M1	M2	M3	M4	M5	M6
**Intercept**	2.634^***^ (0.374)	3.169^***^ (0.289)	3.272^***^ (0.290)	2.215^***^ (0.431)	2.976^***^ (0.513)	3.057^***^ (0.505)
**Control variables**						
*gender*	0.217^*^ (0.106)	0.133 (0.082)	0.119 (0.081)	0.061 (0.123)	0.083 (0.121)	0.125 (0.120)
*age*	-0.018 (0.044)	-0.006 (0.034)	-0.009 (0.034)	-0.016 (0.051)	-0.025 (0.051)	-0.022 (0.050)
*education background*	-0.050 (0.079)	0.014 (0.061)	0.008 (0.060)	-0.103 (0.092)	-0.121 (0.091)	-0.120 (0.089)
*marital status*	0.457^***^ (0.128)	0.276^**^ (0.099)	0.270^**^ (0.098)	-0.152 (0.148)	-0.099 (0.148)	-0.017 (0.148)
*children*	-0.101 (0.083)	-0.127 (0.064)	-0.110 (0.064)	0.157 (0.096)	0.160 (0.095)	0.129 (0.094)
*family month income*	0.031 (0.051)	0.036 (0.039)	0.027 (0.039)	0.033 (0.059)	0.038 (0.058)	0.042 (0.057)
*hospital size*	0.131 (0.069)	0.013 (0.053)	0.009 (0.053)	0.003 (0.079)	0.031 (0.079)	0.043 (0.078)
**IV**						
*Ambidextrous leadership*		0.596^***^ (0.064)	0.572^***^ (0.064)		-0.232^**^ (0.087)	-0.034 (0.108)
**Mediator**						
*Work-to-family enrichment*						-0.277^**^ (0.091)
**Moderator**						
*Work-climate for sharing concerns*		0.271^***^ (0.058)	0.295^***^ (0.058)			
**Interaction**						
*Ambidextrous leadership × Work-climate for sharing concerns*			-0.149^*^ (0.067)			
*Adj R* ^*2*^	0.062	0.452	0.461	0.007	0.018	0.051
*F*	3.30^**^	23.45^***^	21.95^***^	0.75	1.56	2.46^*^

Note: *n* = 265; ^***^p < 0.001, ^**^p < 0.01, ^*^p < 0.05.

Hypothesis 2 predicted the indirect effect of work-to-family enrichment between perceived ambidextrous leadership and mental health. We conducted a bootstrapping method in *PROCESS* of SPSS 21.0. First, perceived ambidextrous leadership was negatively related to mental health disease (Model 5, β=-0.232, *p* = 0.005) and positively related to work-to-family enrichment (Model 2, β = 0.596, *p* = 0.000). The significance of the impact of perceived ambidextrous leadership on mental health disease decreases and becomes nonsignificant (β = -0.034, n.s.) when work-to-family enrichment is entered before the independent variable. In addition, a bootstrapping model was adopted to further test the mediation effect hypothesis. Table 5 indicates that the indirect effect of perceived ambidextrous leadership on mental health disease through work-to-family enrichment was statistically significant because the bias-corrected 95% CI [-0.362, -0.012] excluded zero. Thus, Hypothesis 2 was supported.

**Table 5:** Indirect effects of ambidextrous leadership on mental health disease via work-to-family enrichment

Variables	Estimate	Bootstrapping		
		Bia-corrected 95%CI		
		Lower		Upper
**Total Effect**				
Ambidextrous leadership →mental health disease	-0.180	-0.336	-0.025	
**Direct Effect**				
Ambidextrous leadership →mental health disease	-0.012	-0.210		0.185
**Indirect Effect**				
Ambidextrous leadership →mental health disease	-0.168	-0.362		-0.012

*n* = 265, bootstrapping 2000.

**Fig. 2:**
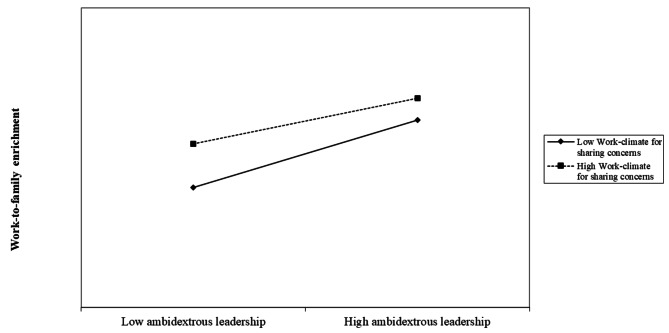
Simple slope test

Hypothesis 3 predicted the moderation effect of work climate for sharing family concerns on the relationship between perceived ambidextrous leadership and work-to-family enrichment. As shown in Table 4, the interaction between perceived ambidextrous leadership and work climate for sharing family concerns was negatively related to work-to-family enrichment (Model 3, β=-0.149, *p* = 0.035). A simple slope test was further conducted. Specifically, work-to-family enrichment was regressed on ambidextrous leadership at high values (mean + 1 sd) and low values (mean-1 sd) of work climate for sharing family concerns. The relationship between perceived ambidextrous leadership and work-to-family enrichment was positive and significant when work climate for sharing family concerns was high (r = 0.462, t = 5.308, p < 0.001). The relationship between perceived ambidextrous leadership and work-to-family enrichment was positive and significant when work climate for sharing family concerns was low (r = 0.682, t = 9.096, p < 0.001). The results showed the slope at low work climate for sharing family concerns was steeper than the slope at high work climate for sharing family concerns. The interaction effect is graphically depicted in Fig. 2. Thus, Hypothesis 3 was supported.

## Discussion

The present study investigated the relationship between perceived ambidextrous leadership and nurses’ mental health via work-to-family enrichment moderated by the work climate for sharing family concerns based on the work-home resources model [26]. Through a time-lagged survey in the hospital, we found that nurses’ perceived ambidextrous leadership was positively related to nurses’ work-to-family enrichment and reduced mental health disease. Work climate for sharing family concerns moderated the relationship between perceived ambidextrous leadership and nurses’ work-to-family enrichment, such that the relationship is stronger when work climate for sharing family concerns is low rather than high.

### Theoretical implications

First, our study provided a constructive perspective, the perspective of work-to-family enrichment, by applying the work-home resources model, to nurses’ mental health literature [26]. Few studies are grounded in the perspective of WFE to investigate nurses’ mental health, even previous studies provide some work-related coping strategies for the high levels of negative mental health that nurses face [63, 64]. This study found that work-to-family enrichment can help alleviate mental health diseases. Work-to-family enrichment helps nurses deal with job difficulties by developing critical psychological and physical resources. This finding is striking because it is notable that there are salient differences in work demand and consequentially health conditions between nurses and salaried employees in other industries. Mark and Smith [63] indicate that nurses are particularly at risk from stress-related problems and face high mental health problems. Work-to-family enrichment can provide a sense of belonging, optimism, self-esteem and confidence [65]. Nurses suffering from mental health diseases can recover by integrating the above psychological resources.

Second, our study advanced the boundary condition in the nursing management context from the conjoint perspective of leadership and climate. Cai et.al. [66] found that ambidextrous leadership could promote clinical leadership and nurses’ work engagement. However, the boundary condition of the effect of ambidextrous leadership has not been investigated. This paper is grounded in social information processing theory and regards colleagues as proximal social cues and leadership as distal social cues. The proximal social cues can substitute for distal social cues. This study found that perceived ambidextrous leadership interacting with a work climate for sharing concerns predicts nurses’ work-to-family enrichment and, sequentially, their mental health. This finding is notable because ambidextrous leadership becomes more effective in the nursing management field under a given climate. This is in accordance with the leadership contingency theory that leadership effectiveness depends on situational control [67]. As Fiedler [67] noted, “a high degree of control and influence implies that the leader has correspondingly high certainty that his decisions and actions will have predictable results and that they will achieve the desired goals and gratify the leader’s needs in the situation” (p. 62). Future studies can investigate the interaction between leadership and other contextual factors (e.g., culture, rules and organizational norms) in the nursing context.

Third, our study contributes to the mechanism of nurses’ mental health. This paper found that perceived ambidextrous leadership is critical for nursing management via enhancing work-to-family enrichment. Previous ambidextrous leadership studies were mostly performed in manufacturing, financial service, and hospitality contexts [58]. Only one study focused on ambidextrous leadership in the nursing context [66]. Ambidextrous leadership can tackle paradoxical problems in organizations [23]. In particular, during COVID-19, nurses faced huge job demands and stress [68]. Nurses are one of the professional groups at the front line of the fight against novel infectious diseases, but they also face substantial tasks and suffer from mental health problems [69]. Ambidextrous leadership can provide workplace support to nurses.

### Practical implications

Workers in health organizations are puzzled by work-family issues due to heavy nursing jobs [70], which results in damage to mental health [6]. Our findings indicated that supervisors’ ambidextrous leadership interacting with the work climate to share concerns could lead nurses to high work-to-family enrichment and improve their mental health status.

First, our study indicated that nursing supervisors can promote nurses’ work-to-family enrichment by implementing ambidextrous leadership. Leadership can not only influence employees’ attitudes and behaviors in the workplace but also have a cross-domain effect on employees’ families through instrumental paths [16]. Specifically, when nursing tasks are routine and general, such as ward rounds and drug storage, nursing supervisors are supposed to clear nurses’ job requirements, design a standard workflow and establish routines. These measures can help nurses perceive predefined job roles, improve working efficiency and conserve cognitive resources [71].

Second, our findings validated the mediating role of work-to-family enrichment between perceived ambidextrous leadership and nurses’ mental health. Thus, effective and feasible measures should be adopted to promote nurses’ work-to-family enrichment. Specifically, nurses can also enhance work-to-family enrichment by adjusting their work attitudes. Research has identified that work involvement, work centrality and work engagement benefit employees’ work-to-family enrichment [72]. That is, nurses are supposed to have a positive attitude toward their work, build emotional connections with organizations and colleagues, and seek meaning and enjoyment within the work role. Being more psychologically invested can not only result in the accumulation of personal valuable resources but also facilitate individuals’ work satisfaction [73]. When nurses maintain a positive mood in the workplace, a positive spillover from the work domain to the family domain occurs, and psychological problems such as anxiety and depression of nurses can also be alleviated.

Third, our findings supported the view that the work climate for sharing family concerns has a substitution effect on how to promote nurses’ work-to-family enrichment. Thus, health organizations should shape a family-friendly work climate for nurses to share family issues. Specifically, nursing supervisors are supposed to allow their subordinates to discuss family issues with colleagues in the workplace. Although conversation with others may not be beneficial for dealing with family problems, it helps the individual release psychological stress and negative emotions [74]. Moreover, nursing supervisors are supposed to encourage nurses to emotionally connect with their partners, which enables them to obtain advice on how to deal with family issues from the partners and then promote work-to-family enrichment.

Fourth, Nursing supervisors can provide nurses with psychological support, including psychological counseling services and mental health education. Supervisors can also organize some activities regularly to improve the communication and cooperation ability among nurses and enhance mutual understanding and support. In addition, supervisors can provide techniques and strategies for coping with stress, such as time management, relaxation training, and emotional regulation techniques.

## Limitations and future research

First, despite a time-lagged survey, all the variables were reported by the nursing staff. The self-report measure may lead to inflated correlations among variables caused by common method bias [75]. Future studies may invite and match both nursing supervisors and nurse staff to rate ambidextrous leadership. Second, nurses’ personality may influence individuals’ mental health. Personality factors (e.g., proactive personality and Big Five personality) should be considered in future research [49]. Third, the majority of our sample is female because most nurse staff are female in China. Future studies may conduct comparable research between males and females. Fourth, we only invited nurses to rate their supervisors’ ambidextrous leadership but did not investigate how supervisors rate the organization managers’ ambidextrous leadership. Future studies may consider “trickle-down” effect of ambidextrous leadership from organization managers to nurses via team supervisors.

## Data Availability

The data can be provided on the request of readers.
